# The New Mayo Clinic Risk Score Characteristics in Acute Coronary Syndrome in Patients Following Percutaneous Coronary Intervention 

**Published:** 2017-10

**Authors:** Lukman Zulkifli Amin, Hilman Zulkifli Amin, Sally Aman Nasution, Marulam Panggabean, Hamzah Shatri

**Affiliations:** Department of Internal Medicine, Faculty of Medicine, Universitas Indonesia, Jakarta, Indonesia.

**Keywords:** *Acute coronary syndrome*, *Percutaneous coronary intervention*, *Mortality*

## Abstract

**Background: **Mortality and major adverse cardiac events (MACE) frequently occur after percutaneous coronary intervention (PCI). Therefore, the ability to predict such events through an established risk stratification method is of great importance. The present study was aimed at determining the risk stratification of mortality and MACE in post-PCI patients at the intensive cardiac care unit of Cipto Mangunkusumo Hospital (CMH) using 7 variables of the New Mayo Clinic Risk Score (NMCRS).

**Method: **This cross-sectional study drew upon secondary data gathered from the medical records of 313 patients that underwent PCI at the intensive cardiac care unit (ICCU) of CMH between August 1st, 2013, and August 31st, 2014. The primary end point was all-cause mortality and MACE. Seven variables in the NMCRS, namely age, left ventricular ejection fraction, serum creatinine, preprocedural cardiogenic shock, myocardial infarction, and peripheral arterial disease, were evaluated.

**Results:** The mortality and MACE incidence rates in the post-PCI patients were 3.8% (95%CI: 2.6-5.0) and 8.3% (95% CI: 6.6-10.0), respectively. Regarding the NMCRS stratification, elderly patients with lower left ventricular ejection fraction, increased serum creatinine, preprocedural cardiogenic shock, myocardial infarction, and peripheral arterial disease had higher mortality and MACE incidence rates among the post-PCI patients. The mortality and MACE incidence rates significantly increased in the post-PCI patients with a higher NMCRS.

**Conclusion:** Patients with a higher NMCRS had a tendency toward higher mortality and MACE incidence rates following PCI.

## Introduction

Coronary heart disease is the main cause of death in developed and developing countries, including Indonesia. The medical treatment strategy of patients with coronary heart disease has developed rapidly. That advancement includes therapy to control its risk factors, types of thrombolytic drugs, and coronary intervention.^1^^, ^^2^ According to the American Heart Association, coronary heart disease was culpable for 1 in every 7 deaths in the United States in 2011. In addition, Riset Kesehatan Dasar (Riskesdas) in 2013 showed that coronary heart disease was the third leading cause of death in Indonesia. Moreover, acute myocardial infarction was the second leading cause of death in low-income countries, with a mortality rate of 2.47 million (9.4%) from 25.5 million deaths, as was cited by the Simarmata et al. study at Universitas Sumatera Utara in 2011.

Intervention becomes an option when coronary angiography reveals obstruction in the coronary arteries. Percutaneous coronary intervention (PCI) is a nonsurgical coronary revascularization modality that may be urgent or elective. This intervention has complications that could lead to death and major adverse cardiac events (MACE), including recurrent myocardial infarction and stroke. Research also shows that in-hospital recurrent revascularization occurs in 8%-10% of patients with the acute coronary syndrome.^3^

Risk stratification is used by physicians in an attempt to predict complications in the wake of PCI. Post-PCI risk stratification is an important factor to optimize the management of post-PCI patients. This risk prediction model can help healthcare professionals to assess the risk of complications after conducting PCI. The New Mayo Clinic Risk Score (NMCRS) has been widely known as a risk score tool for predicting mortality events after PCI. The present study employed the NMCRS as its scoring system because its 7 variables are easily obtainable, making it possible to assess mortality risk before PCI. The NMCRS is a mortality and MACE risk prediction model in post-PCI patients with demographic variables of age, serum creatinine level, left ventricular ejection fraction, myocardial infarction within 24 hours or less, preprocedural cardiogenic shock, chronic heart failure, and peripheral arterial disease.^4^

The current study was conducted at the intensive cardiac care unit (ICCU) of Cipto Mangunkusumo Hospital (CMH) to determine not only the incidence of mortality and MACE but also the risk of mortality and MACE in post-PCI patients in the ICCU based on the NMCRS stratification for this group of patients with a view to determining the potency of the NMCRS as a prognostic score tool. 

## Methods

This was a cross-sectional study to determine the incidence and risk of mortality and MACE based on the NMCRS stratification in post-PCI patients admitted to the ICCU of CMH. We drew upon secondary data obtained from the medical records of patients who underwent PCI in the ICCU of CMH. Data were taken from patients admitted between August 1st, 2013, and August 31st, 2014. The target population and research subjects comprised patients with the acute coronary syndrome that underwent PCI in the ICCU of CMH. Only patients with complete NMRCS variables stated in the medical records of the ICCU of CMH were included in the study, and patients with incomplete data were excluded. For the statistical analyses, the statistical software SPSS version 17.0 for Windows (SPSS Inc., Chicago, IL) was used. The data are described for the categorical variables in percentages, and the numerical data are presented in means ± SDs. The study protocol was approved by the Research Legal and Ethics Committee of CMH and the Faculty of Medicine, Universitas Indonesia.

## Results

A total of 313 patients were included in the present study, and 87 patients with incomplete medical records were excluded. The study population was at a mean age of 59.97 ± 10.62 years. The top NMCRS characteristic was myocardial infarction within 24 hours or less, while the bottom NMCRS characteristic was peripheral arterial disease. 

**Table 1:** Characteristics of the subjects (n=313)*

Age (y)	59.97±10.62
Sex	
Male	227 (72.5)
Female	86 (27.5)
Unstable Angina	108 (34.5)
MI within 24 hours or less	177 (56.5)
Preprocedural cardiogenic shock	35 (11.2)
Chronic heart failure	53 (16.9)
Pre-PCI serum creatinine level (mg/dL)	1.67±2.04
LVEF (%)	52.33±15.87
Hypertension	217 (69.3)
Diabetes mellitus	110 (35.1)
PAD	9 (2.9)

MI, Myocardial infarction; PCI, Percutaneous coronary intervention; LVEF, Left ventricular ejection fraction; PAD, Peripheral arterial disease

* Data are presented as mean±SD or n (%).

The characteristics of the subjects, at an average age of 59.97 ± 10.62 years, are shown in Table 1. Males constituted the dominant population. Unstable angina was more frequent than myocardial infarction. Other related conditions such as preprocedural cardiogenic shock, chronic heart failure, hypertension, and diabetes mellitus are also described in the table.

The incidence rates of mortality and MACE in the post-PCI patients during the treatment reached 3.8% (95% CI: 2.6–5.0) and 8.3% (95% CI: 6.6–10.0), respectively. In addition, an increase in age and the serum creatinine level was in tandem with a rise in the rates of mortality and MACE following PCI.

Table 2 shows that elderly age, unstable angina pectoris, myocardial infarction within 24 hours or less, preprocedural cardiogenic shock, LVEF, chronic heart failure, serum creatinine, and peripheral arterial disease were correlated with an increase in mortality and MACE incidence.

The higher NMCRS mortality risk category was correlated with the increase in mortality incidence, as is shown in Table 3.

Table 4 shows that the higher NMCRS MACE risk category was correlated with an increase in MACE incidence.

**Table 2:** Patients’ mortality and MACE based on the new Mayo Clinic risk score (NMCRS) characteristics (n=313)

Characteristics	Number of Cases	Mortality		MACE	
		Number	% (95% CI )	Number	% (95% CI )
Age (y)					
30-38	25	0	0	0	0
39-48	49	1	2.0 (1.88-2.20)	3	6.1 (5.50-6.70)
49-66	80	3	3.8 (3.40-4.10)	9	11.3 (10.07-12.43)
67-73	112	4	3.6 (3.24-3.90)	9	8.03 (7.20-8.86)
74-79	47	4	8.5 (7.63-9.39)	5	10.6 (9.51-11.75)
79-87	0	0	0	0	0
88	0	0	0	0	0
Unstable angina pectoris	108	2	1.8 (1.66-1.94)	7	6.5 (5.90-7.10)
Myocardial infarction	205	10	4.9 (4.42-5.38)	19	9.3 (8.29-10.25)
Myocardial infarction within 24 hours or less	177	9	5.0 (4.51-5.49)	16	9.0 (8.10-9.90)
Myocardial infarction after 24 hours	28	1	3.6 (3.27-3.93)	3	10.7 (9.60-11.80)
Preprocedural cardiogenic shock	35	6	17.1 (15.26-18.94)	10	28.6 (25.50-31.70)
No preprocedural cardiogenic shock	278	6	2.2 (1.63-2.77)	16	5.8 (5.18-6.32)
Left ventricular ejection fraction (%)					
≥ 60	129	1	0.8 (0.76-0.84)	8	6.2 (5.60-6.80)
40-59	111	6	5.4 (4.86-5.94)	9	8.1 (7.30-8.90)
20-39	65	3	4.6 (4.15-5.05)	7	10.8 (9.70-11.90)
< 20	8	2	25.0 (21.25-28.75)	2	25.0 (21.25-28.75)
Chronic heart failure	53	2	3.8 (3.44-4.16)	5	9.4 (8.50-10.30)
No chronic heart failure	260	10	3.8 (3.44-4.16)	21	8.1 (7.24-8.90)
Serum creatinine (mg/dL)					
< 0.60	39	1	2.6 (2.38-2.82)	3	7.7 (6.90-8.50)
0.60-1.29	87	2	2.3 (2.11-2.47)	6	6.9 (6.19-7.59)
1.30-1.49	81	2	2.5 (2.26-2.66)	6	7.4 (6.64-8.16)
1.50-1.99	45	3	6.7 (6.02-7.38)	5	11.1 (9.90-12.30)
2.00-3.99	17	0	0	0	0
≥ 4.00	64	4	6.3 (5.62-6.88)	6	9.4 (8.40-10.36)
Peripheral arterial disease	9	2	22.2 (19.80-24.60)	1	11.1 (9.90-12.30)
No peripheral arterial disease	304	10	3.29 (2.79-3.59)	25	8.2 (7.40-10.0)

MACE, Major adverse cardiac events

**Table 3:** Patients’ mortality in each the new Mayo Clinic risk score (NMCRS) mortality risk category

NMCRS Mortality Risk Category	Number of Subjects	Mortality	
		Number	**%** (95% CI)
Very low	167	2	1.2 (1.15-1.25)
Low	60	0	0
Moderate	47	2	4.3 (3.85-4.60)
High	10	1	10.0 (8.95-11.05)
Very high	29	7	24.1 (21.53-26.70)

**Table 4:** Major adverse cardiac events (MACE) in each the new Mayo Clinic risk score (NMCRS) MACE risk category

NMCRS MACE Risk Category	Number of Subjects	MACE	
		Number	% (95% CI )
Very low	101	4	4.0 (3.59-4.33)
Low	128	7	5.5 (5.19-5.73)
Moderate	52	4	7.7 (6.91-8.47)
High	16	5	31.3 (27.85-34.65)
Very high	16	6	37.5 (33.50-41.50)

## Discussion

The present cross-sectional study was conducted on 313 subjects with characteristics similar to those in other studies. The median age of the study population was 59.97 ± 10.62 years, which is in line with another study that reported a similar average age of 60.4 years.^5^ Our results showed a rise in the incidence of mortality and MACE after PCI in association with increased age. Those in the age bracket of 74-79 years had the most mortality events (8.56%) of all age groups (95%CI: 7.6-9.4). Many other studies, including that of Moeswir et al. at Universitas Indonesia in 2014, have also revealed that increased age is significantly associated with increased mortality and MACE after PCI.^6^-^9^ Annika et al. revealed that the rate of MACE in their elderly population reached 5% and that there was an increase in the mortality risk of the elderly patients (> 65 y) (OR = 3.54, 95%CI: 2.4-5.3).^10^ The elderly tend to have a higher risk of mortality and MACE after PCI because they are more likely to have other comorbidities such as diabetes mellitus and hypertension. 

Thirty-five percent of our study population had unstable angina, with the rate of myocardial infarction within 24 hours or less amounting to 57%. A previous study reported corresponding rates of 54% and 49%.^4^ Our results demonstrated that the rates of mortality and MACE after PCI in patients with unstable angina were 1.8% (95%CI: 1.7–1.9) and 6.5% (95%CI: 5.9–7.1). Previous studies, including that of Setyawan et al. at Universitas Indonesia in 2013), have also shown high rates of mortality in patients with unstable angina that underwent PCI compared to our study.^11^, ^12^ This could be the result of different characteristics and comorbidities in other studies. 

Myocardial infarction in our study had a mortality rate of 4.9% (95%CI: 4.4–5.4) and a MACE rate of 9.3% (95%CI: 8.3–9.3); both of these rates are higher than those in our patients with unstable angina. It can be concluded that myocardial infarction could lead to a higher risk of mortality and MACE after PCI.

In addition, our results showed that patients with preprocedural cardiogenic shock had a mortality incidence rate of 17.1% and a MACE incidence rate of 28.6% after PCI. In other words, approximately 1 out of 10 patients and 1 in 5 patients with preprocedural cardiogenic shock were likely to experience mortality and MACE post PCI. On the other hand, non-preprocedural cardiogenic shock patients had post-PCI mortality and MACE incidence rates of 2.2% and 5.8%, respectively. These results indicated that cardiogenic shock could lead to higher risk of mortality and MACE after PCI. Preprocedural cardiogenic shock caused a decrease in cardiac output, resulting in hypotension and reduced coronary and systemic perfusion. This in turn triggered vasoconstriction reflex as well as systemic inflammation, causing more severe myocardial dysfunction, mortality, and MACE.^11^ These results chime in with those of previous studies revealing that preprocedural cardiogenic shock was correlated with a higher risk of mortality and MACE after PCI.^12^, ^13^

Our results also demonstrated that low left ventricular ejection fraction was associated with a higher risk of mortality and MACE after PCI. Patients with ejection fraction < 20% had a 25% (95%CI: 21.3–28.8) rate of mortality and MACE following PCI. This result is concordant with that in a study in the United Kingdom, which reported that abnormal left ventricular function was an independent predictor of 30-day mortality after PCI. Thirty-day mortality risk had a hazard ratio of 2.91 (95%CI: 2.4-3.5; p value < 0.0001) for patients with moderate left ventricular function (ejection fraction = 30%-49%) and a hazard ratio of 7.3 (95%CI: 5:9-9:0; p value < 0.0001) for patients with low left ventricular function (ejection fraction < 30%).^14^ Chronic heart failure also exhibited a tendency toward an elevated risk of mortality and MACE after PCI. Our study revealed that patients with chronic heart failure had a mortality rate of 3.8% (95% CI: 3.4-4.2) and a MACE rate of 9.4% (95% CI: 8.5-10.3). 

Our results demonstrated that a rise in the serum creatinine level was associated with a higher likelihood of increased mortality and MACE after PCI. Patients with a serum creatinine level of 1.5-1.9 mg/dL had a higher incidence of mortality (6.5%; 95%CI: 6.0-7.4) and MACE (11.1%; 95%CI: 9.9-12.3) following PCI. This finding is consistent with the result of another study, which showed that renal function was independently associated with 30-day mortality (HR = 1.6, 95%CI: 1.2-2.1; p value = 0.003). Additionally, the authors of that study reported that in chronic kidney disease patients with a glomerular filtration rate < 60 mL/min / 1.73 m2, the incidence of MACE was more often comparable to the rate in those without chronic kidney disease (15% and 4%). Nevertheless, the incidence of 30-day mortality was higher in their patients with chronic kidney disease than in those without it (9% and 2%).^15^ Furthermore, patients with peripheral arterial disease also showed similar tendency in increasing mortality risk and MACE after PCI. A study by Wood et al., published in Heartwire: Medscape News in 2002, stated that within 1 year, patients with a history of peripheral vascular disease had a mortality rate 2.3 times greater than that of those without it (p value < 0.001). Elsewhere, Keeley^16^ reported an incidence rate of 13% for MACE following PCI among 1062 patients with peripheral arterial disease.

Based on the NMCRS stratification, our study revealed that patients in the higher-risk group had a tendency toward an increase in mortality and MACE incidence after PCI. We also found that patients classified in the “very-high” risk category of the NMCRS had mortality and MACE rates of 24.1% and 37.5%, correspondingly, following PCI. Singh et al.^4^ also showed similar results with respective rates of 27.2% and 37.8% for mortality and MACE subsequent to PCI in the “very-high” risk category of the NMCRS. It can be concluded that an increase in the NMCRS is correlated with a rise in the incidence of mortality and MACE after PCI and, thus, can be used as a prognostic tool for predicting post-PCI complications. 

There were several limitations in our study. Eighty-seven medical records (27.8%) from a total of 313 were incomplete for the NMCRS variables. In addition, the data taken tended to be subjective in that they were based only on what was already written in the medical records. Furthermore, the present study cannot be used for scoring validation purposes due to its limited sample numbers. 

## Conclusion

The incidence rates of mortality and MACE after PCI in the ICCU of CMH were 3.8% (95%CI: 2.6–5.0) and 8.3% (95%CI: 6.6–10.0), respectively. Based on the NMCRS stratification, our study revealed that patients in the higher-risk group had a tendency toward an increase in the incidence rates of mortality and MACE in the wake of PCI. A rise in the NMCRS was in line and associated with a rise in the incidence of mortality and MACE after PCI; it can, therefore, be utilized as a prognostic tool for the prediction of post-PCI complications. The NMCRS as a simple risk-stratification and prognostic scoring tool for evaluating post-PCI complications has a good performance. Further larger studies are, however, needed to validate the NMCRS in terms of feasibility and applicability vis-à-vis each specific patient characteristic. 

## Notes:

***This paper should be cited as:*** Zulkifli Amin L, Zulkifli Amin H, Aman Nasution S, Panggabean M, Shatri H. The New Mayo Clinic Risk Score Characteristics in Acute Coronary Syndrome in Patients Following Percutaneous Coronary Intervention. J Teh Univ Heart Ctr 2017;12(4):149-154.
